# The relationship between glucose homeostasis status and prostate size in aging Chinese males with benign prostatic hyperplasia

**DOI:** 10.1007/s00345-020-03084-4

**Published:** 2020-01-21

**Authors:** Y. Wu, Y. Ding, Q. F. Cao, S. B. Qian, C. Wang, H. Q. Duan, J. Gu, H. B. Shen

**Affiliations:** Department of Urology, Xinhua Hospital, School of Medicine, Shanghai Jiaotong University, 1665 Kongjiang Road, Shanghai, 200092 China

**Keywords:** Benign prostate hyperplasia, Glucose homeostasis, Prediabetes, Prostate size

## Abstract

**Purpose:**

Increasing evidence shows that many metabolic factors are involved in the progression of benign prostatic hyperplasia (BPH). We aimed to assess the relationship between the status of glucose homeostasis and prostate size in aging Chinese males undergoing transurethral resection of the prostate (TURP) for BPH.

**Methods:**

A total of 1006 medical records of BPH patients undergoing TURP were reviewed. Prostate size was measured by transrectal ultrasound. Annual total prostate (TP) and transitional zone (TZ) growth rates were calculated. According to the American Diabetes Association criteria, the patients were categorized as normoglycemic, prediabetic, or diabetic. Levels of glucose homeostasis and other variables were considered independent variables in an effort to evaluate any potential correlations using non-adjusted and multivariate-adjusted regression models.

**Results:**

A total of 659 individuals were included in the study. BPH patients < 70 years old and ≥ 70 years old in the normoglycemic group had a stable prostate growth rate. The change in prostate size in those younger than 70 years, however, was faster in the prediabetic and diabetic group. Further analysis revealed that abnormal glucose homeostasis was positively correlated with prostate size. In those younger than 70 years, compared with the normal glucose group, the adjusted odds ratio (OR) for TP and TZ enlargement in the prediabetic group was 2.27 (95%CI 1.29–4.00) and 3.19 (95%CI 1.78–5.72), respectively, and the adjusted ORs were 4.74 (95%CI 2.18–10.30) and 6.16 (95%CI 2.70–14.06), respectively, for men with diabetes. However there was no significant difference among men aged ≥ 70 years.

**Conclusions:**

Among patients undergoing TURP, the prostate volume and growth rate were affected by different status of glucose homeostasis. Hyperglycemia may play an important role in prostate growth.

**Electronic supplementary material:**

The online version of this article (10.1007/s00345-020-03084-4) contains supplementary material, which is available to authorized users.

## Introduction

Benign prostatic hyperplasia (BPH) is a common disease in middle-aged and elderly men. Secondary lower urinary tract symptoms (LUTS) have a serious impact on quality of life. Histologically, BPH is a nonmalignant, unregulated proliferation of stromal and epithelial prostate cells. Recent studies have found that metabolic diseases such as obesity and diabetes are involved in the progression of BPH [1, 2]. Since the age of onset of diabetes is similar to that of prostatic hyperplasia, the influence of blood glucose and related factors on the prostatic gland has received increasing attention. The increase in prostate size is one of the research hotspots, because the size of the prostate is related to the prevalence of LUTS and BPH surgery [3, 4] and many studies have found that diabetes was significantly associated with increased prostate volume [1, 5, 6]. Prediabetes is related to diabetes, but they are not exactly the same. Prediabetes is the precursor stage before diabetes and has been viewed as an increased risk factor for diabetes and cardiovascular disease [7]. The prevalence of prediabetes also increases with age. It was 38% in the U.S. in 2011–2012 [8] and 35.7% in China in 2013 [9]. However, despite the large number of patients, prediabetes has not been studied, and it is not yet clear whether prediabetes also has an effect on prostate size.

Aging is also a significant causative factor in the development of BPH [10]. Many studies have confirmed that prostate volume increases with age [11, 12]. Moreover, in recent years, some studies have pointed out that the prostate growth rate before the age of 70 is significantly faster and more stable than after the age of 70 [13–15]. Although both diabetes and age are associated with prostate volume growth, it is not clear whether there is an effect or synergistic amplification between the two. In addition, the effects of aging on prostate size enlargement and changes in prostate growth before and after the age of 70 are worth investigating in groups of patients with different glucose homeostasis statuses.

Therefore, we conducted a retrospective study to evaluate the possible association of glucose homeostasis status, age and prostate size in BPH patients who underwent prostate surgery.

## Materials and methods

### Study design and participants

We retrospectively reviewed the clinical data of patients treated for symptomatic BPH/LUTS (symptoms of urinary storage, voiding, and late voiding) in our department from December 2012 to December 2017. A total of 1006 patients had received transurethral resection of the prostate (TURP). The indications for TURP were as follows: recurrent urinary retention, recurrent urinary tract infections, recurrent macroscopic hematuria, bladder stones or diverticula, dilation of the upper urinary tract with or without renal insufficiency, and patient’s will or request [16]. The general characteristics of the patients included age, height, weight, usage of medications, time of admission, medical history and the following comorbidities: hypertension, coronary disease, liver disease, diabetes, neuropathy, and tumors of any type. To minimize potential confusion and bias, we excluded 347 patients with a surgical history of BPH, urinary malignancies, neurogenic bladder, prostate cancer, liver cirrhosis, or obvious neuropathy; we also excluded men who take hormones, antiandrogen agents, antifungal agents, steroidal agents, or 5-α reductase inhibitor (5ARI) in the last 6 months. A total of 659 patients were ultimately included in the analytic sample.

### Data collection

Serum blood samples were drawn between 6:00 AM and 8:00 AM after overnight fasting for at least 8 h. Levels of fasting plasma glucose (FPG), glycated hemoglobin (HbA1c) and blood lipids were measured using standard laboratory techniques at our hospital. The total testosterone (TT) concentrations were detected by electrochemiluminescence immunoassay.

Prostate size parameters, including total prostate volume (TPV) and transitional zone volume (TZV) were determined by transrectal ultrasound (Siemens Sequoia 512, linear array probe 15L8W, frequency 3–8 MHz). Images were obtained with the patient in the left decubitus position. We determined the TPV using the formula for a prostate ellipsoid: width × length × height × (*π*/6). Width was measured as the largest section on the transverse scan, length as the greatest anteroposterior distance on the sagittal scan, and height as the longest cephalad-to-caudal dimension in the sagittal plane. The prostate transitional zone volume was also calculated using the above formula.

Body mass index (BMI) was calculated by weight in kilograms divided by height in meters squared (kg/m^2^). The prostate transition zone index (TZI) is the ratio of TZV to TPV. Annual TPV and TZV growth rates were calculated by the following formula: annual TPV growth rate = (TPV−20) mL/(age−40) years; annual TZV growth rate = TZV mL/(age – 40) years [17]. All data were measured by two experienced doctors.

### Grouping criteria

According to standards established by the American Diabetes Association (ADA), patients with prediabetes were defined as any participants who did not have diabetes but who had an HbA1c level of 5.7– 6.4%, a FPG level of 100–125 mg/dL (5.6 to 7.0 mmol/L) or a 2-hour plasma glucose level of 140–199 mg/dL (7.8 to 11.1 mmol/L) [18]. Participants were divided into three categories: normoglycemic (FPG was under 5.6 mmol/L or HbA1c was under 5.7%), prediabetic (FPG was between 5.6 mmol/L and 7.0 mmol/L or HbA1c was between 5.7% and 6.4%), and diabetic (FPG was over 7.0 mmol/L or HbA1c was over 6.4%). The patients with a history of diabetes were directly classified into the diabetic group. We defined BMIs in the range of 24.0–28.0 kg/m^2^ as overweight and BMI ≥ 28.0 kg/m^2^ as obesity according to Chinese adult standards. (National Health Commission of the People’s Republic of China. Criteria of Weight for Adults 2013, https://www.nhc.gov.cn/ewebeditor/uploadfile/2013/08/20130808135715967). Additionally, according to the BMI distribution, the patients were divided into two groups: the normal BMI group (BMI < 24 kg/m^2^) and the abnormal BMI group (BMI ≥ 24 kg/m^2^).

### Statistical analysis

The statistical analysis was performed using SPSS Statistics version 21.0 (SPSS Inc., Chicago, IL). Data were expressed as the mean (standard deviation, SD) or median (interquartile range) for continuous variables depending on the data distribution. Multiple imputation (SPSS-based EM method) was used to generate new data and process missing data. Normality plots with tests and homogeneity of variance were used to determine whether the distribution of effect sizes was symmetrical between groups. The differences between groups were evaluated by Student’s *t *test, Mann–Whitney *U* test or Kruskal–Wallis rank sum test for continuous variables. Linear and logistic regressions were performed to evaluate the relationship between the three different glycemic status groups and prostate size. Both non-adjusted and multivariate-adjusted models were applied for potential confounders. Variables such as age, BMI, TT, total cholesterol (TC), triglycerides (TG), high-density lipoprotein cholesterol (HDL-C) and low-density lipoprotein cholesterol (LDL-C) were taken into account and analyzed continuously. Variables that changed the effect value by more than 10% were layered for further subgroup analysis. A two-tailed *P* < 0.05 was considered statistically significant.

## Results

A total of 659 BPH patients were ultimately included in our study. The median age was 71 years. The prevalence rates of prediabetes and diabetes were 37.2% (245/659) and 23.8% (157/659) in the overall study population, respectively. The baseline clinical characteristics of the three groups are shown in Table 1. Prostate growth rate and prostate volume, including total volume and transition zone volume, increased with glycemic status (Table 1).

**Table 1:** The baseline characteristics of patients with BPH expressed as median with interquartile (*n* = 659)

Variables	Total (*n* = 659)	Normal (*n* = 257, 39.0%)	Pre-diabetic (*n* = 245, 37.2%)	Diabetic (*n* = 157, 23.8%)	*P* value
Age (years)	71 (66–78)	70 (66–78)	72 (66–78)	73 (68–80)	< 0.001
BMI (kg/m^2^)	24.2 (21.8–26.2)	23.5 (21.5–25.8)	23.9 (21.7–26.0)	25.2 (22.7–27.0)	< 0.001
FPG (mmol/L)	5.2 (4.7–5.9)	4.9 (4.6–5.1)	5.2 (4.8–5.7)	6.9 (5.9–7.9)	< 0.001
HbA1c (%)	5.8 (5.5–6.2)	5.4 (5.2–5.5)	5.9 (5.7–6.0)	7.0 (6.5–7.9)	< 0.001
TC (mmol/L)	4.3 (3.8–4.8)	4.3 (3.8–4.8)	4.3 (3.8–4.8)	4.3 (3.7–5.00)	0.904
TG (mmol/L)	1.2 (0.8–1.6)	1.2 (0.7–1.6)	1.2 (0.8–1.5)	1.2 (0.8–1.9)	0.244
HDL-C (mmol/L)	1.2 (1.1–1.5)	1.3 (1.1–1.5)	1.2 (1.1–1.5)	1.2 (1.0–1.3)	0.002
LDL-C (mmol/L)	2.4 (2.1–2.8)	2.4 (2.1–2.8)	2.5 (2.1–2.8)	2.4 (2.1–3.0)	0.696
TT (mmol/L)	12.7 (10.6–15.0)	13.3 (11.4–15.4)	12.7 (10.4–15.3)	11.8 (9.8–13.8)	< 0.001
TP volume (mL)	67.0 (49.0–88.0)	59.0 (43.1–81.0)	67.0 (51.9–90.6)	78.0 (57.3–96.7)	< 0.001
TZ volume (mL)	40.0 (24.0–58.4)	33.0 (18.6–52.3)	40.3 (26.0–61.9)	48.0 (31.6–65.3)	< 0.001
TZI	0.60 (0.48–0.68)	0.57 (0.41–0.67)	0.60 (0.51–0.69)	0.64 (0.53–0.70)	< 0.001
Annual TP growth rate (mL/year)	1.48 (0.94–2.24)	1.36 (0.80–2.04)	1.49 (1.00–2.29)	1.73 (1.14–2.53)	0.001
Annual TZ growth rate (mL/year)	1.25 (0.81–1.82)	1.15 (0.69–1.59)	1.26 (0.83–1.89)	1.42 (0.97–2.23)	< 0.001

Linear regression analysis revealed that the different glycemic statuses were positively correlated with prostate size in the non-adjusted model. Compared with the normoglycemic group, TPV in the prediabetes and diabetes groups was significantly increased by 8.34 mL and 18.33 mL respectively; TZV in the prediabetes and diabetes groups increased by 7.57 mL and 15.35 mL, respectively; and TZI in the prediabetes and diabetes groups increased by 0.05 and 0.07, respectively. After adjusting for age, BMI, TT TC, TG, HDL-C and LDL-C, statistically significant results remained (Table 2). Additionally, a change of effect value over 10% was only observed for age, and a stratification analysis of age was carried out. This further analysis showed a different result. Among men < 70 years old, the prostate size parameters in the abnormal blood glucose groups were still higher than those in the normoglycemic group, regardless of whether variables were adjusted. Among the men ≥ 70 years old, only the diabetic group had a significant increase in prostate TPV and TZV, while there was no obvious difference in the the prediabetes group (Table 2).

**Table 2:** The linear regression analysis of different glucose homeostasis and prostate volume

	*n*	*β* (95%CI)	*P* value	Model β (95%CI)^a^	*P* value	*n*	*β* (95%CI)	*P* value	Model ^b^ *β* (95%CI)	*P* value	*n*	*β* (95%CI)	*P* value	Model *β* (95%CI)^b^	*P* value
		Total				Age < *70*					Age*** ≥ ****70*				
TPV															
Normal	257	Ref		Ref		125	Ref		Ref		132	Ref		Ref	
Pre-diabetic	245	8.34 (2.95, 13.73)	0.002	5.89 (0.47, 11.30)	0.033	96	11.46 (4.59, 18.32)	0.001	11.36 (4.73, 18.00)	0.001	149	4.38 (-3.45, 12.22)	0.272	2.70 (-5.35, 10.73)	0.511
Diabetic	157	18.33 (12.21, 24.44)	0.000	13.56 (7.21, 19.91)	0.000	50	18.53 (10.06, 27.00)	0.000	16.13 (7.62, 24.65)	0.000	107	15.14 (6.62, 23.67)	0.001	11.53 (2.55, 20.51)	0.012
TZV															
Normal	257	Ref		Ref		125	Ref		Ref		132	Ref		Ref	
Pre-diabetic	245	7.57 (3.01, 12.13)	0.001	5.05 (0.49, 9.61)	0.030	96	10.03 (4.07, 15.99)	0.001	10.06 (4.31, 15.81)	0.001	149	4.12 (-2.41, 10.64)	0.215	2.18 (-4.51, 8.86)	0.522
Diabetic	157	15.35 (10.18, 20.51)	0.000	11.11 (5.76, 16.47)	0.000	50	16.48 (9.13, 23.82)	0.000	14.52 (7.14, 21.90)	0.000	107	11.85 (4.76, 18.95)	0.001	8.62 (1.15, 16.08)	0.024
TZI															
Normal	257	Ref		Ref		125	Ref		Ref		132	Ref		Ref	
Pre-diabetic	245	0.05 (0.02, 0.07)	0.000	0.03 (0.01, 0.06)	0.016	96	0.07 (0.03, 0.11)	0.001	0.07 (0.03, 0.11)	0.001	149	0.02 (-0.01, 0.05)	0.192	0.01 (-0.03, 0.04)	0.756
Diabetic	157	0.07 (0.04, 0.10)	0.000	0.05 (0.02, 0.08)	0.001	50	0.10 (0.05, 0.15)	0.000	0.10 (0.05, 0.15)	0.000	107	0.03 (-0.00, 0.07)	0.081	0.02 (-0.02, 0.05)	0.399

^a^The model was adjusted by age, body mass index, total testosterone, total cholesterol, triglyceride, high-density lipoprotein cholesterol and low-density lipoprotein cholesterol

^b^The model was adjusted by body mass index, total testosterone, total cholesterol, triglyceride, high-density lipoprotein cholesterol and low-density lipoprotein cholesterol

**Fig. 1:**
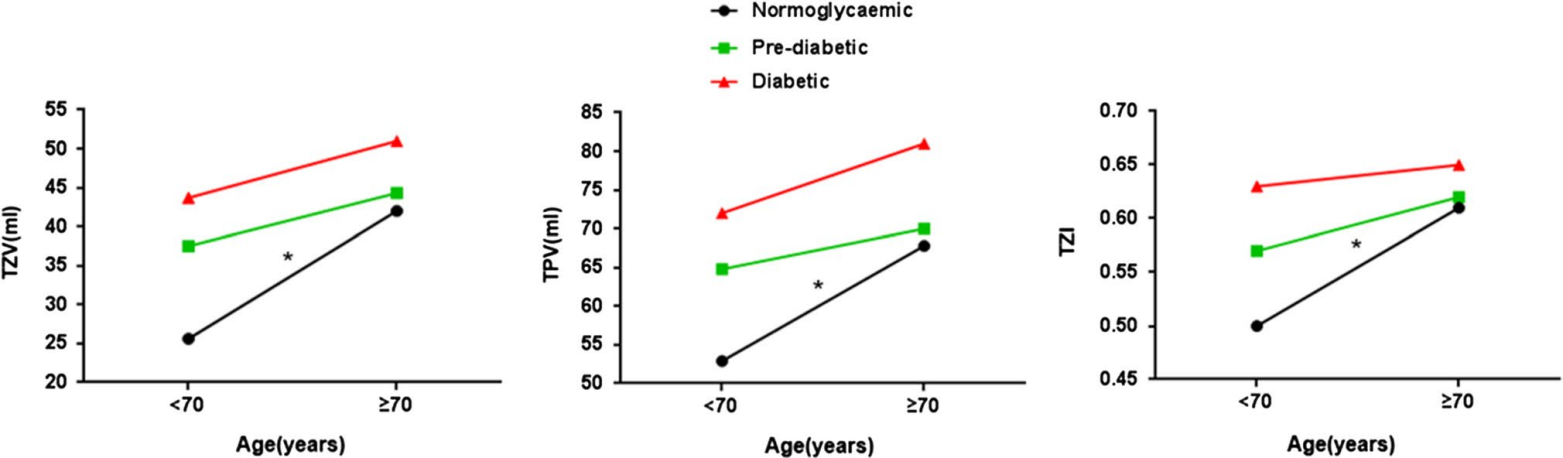
The prostate volume of BPH patients in different blood glucose status groups before and after 70 years old. *Significant difference (*P* < 0.05)

We divided the patients into two subgroups at the age of 70 and compared their prostate parameters in the three different blood glucose states. The data further showed that BPH patients ≥ 70 years old had significantly larger prostate volumes than those < 70 years old in the normoglycemic group (Fig. 1). However, the annual growth rate remained stable in those < 70 years old and in those ≥ 70 years old (*P* > 0.05). Although there were no significant changes in prostate size in either age group by blood glucose abnormality groups, the prostate growth in the men < 70 was faster in the prediabetes group and the diabetic group (Table 3).

**Table 3:** The comparison of prostate morphology in different glucose status between the group with age < 70 and the group with age ≥ 70

Groups	Normal			Pre-diabetic			Diabetic		
Variables	Age < 70	Age ≥ 70	*P*	Age < 70	Age ≥ 70	*P*	Age < 70	Age ≥ 70	*P*
*N* of men	125	132		96	149		50	107	
Age (year)^a^	65(60–68)	76.5(72.0–81.0)		66(63–67)	77(73–81)		67(64–68)	77(73–82)	
TP volume (ml) ^a^	52.9(40.5–71.2)	67.8(50.0–84.0)	< 0.001	64.8(50.0–85.4)	70.0(52.9–92.3)	0.156	72.0(56.7–92.2)	81.0(60.0–102.0)	0.209
Annual TP growth rate (ml/year) ^a^	1.39(0.81–2.43	1.31(0.80–1.75)	0.069	1.79(1.21–2.55)	1.28(0.87–2.04)	< 0.001	2.21(1.46–2.84)	1.61(0.98–2.25)	0.008
TZ volume, ml ^a^	25.6(16.4–40.9)	42.0(23.6–57.7)	< 0.001	37.5(25.0–59.3)	44.3(27.2–63.2)	0.099	43.7(30.4–62.2)	51.0(32.0–71.0)	0.237
Annual TZ growth rate (ml/year) ^a^	1.17(0.70–1.77)	1.14(0.63–1.50)	0.281	1.47(0.97–2.39)	1.10(0.75–1.77)	0.001	1.71(1.24–2.38)	1.33(0.93–1.79)	0.002
TZI ^a^	0.50(0.37–0.64)	0.61(0.50–0.68)	< 0.001	0.57(0.48–0.68)	0.62(0.53–0.70)	0.068	0.63(0.52–0.71)	0.65(0.54–0.70)	0.616

^a^Median with interquartile for continuous variables

According to the prostate size distribution, the thresholds of TPV, TZV and TZI were set at 60 mL, 30 mL and 0.5, respectively. We converted these prostate parameters into categorical variables. Logistic regression found that the men in both glucose abnormality groups had a higher risk of a large prostate compared to the men in the normal group before and after adjusting for age, BMI, TT TC, TG, HDL-C and LDL-C. After dichotomizing age at 70 years old, the data showed that the above trend still existed among the men aged less than 70 years, but there was no significant difference among the men ≥ 70 years old (Table 4). In addition, according to the distribution of BMI, patients were divided into normal BMI (BMI < 24 kg/m^2^) or abnormal BMI (BMI ≥ 24 kg/m^2^) groups. The data showed that there was no significant difference between these two subgroups (Supplementary Table 1).

**Table 4:** The logistic regression analysis of risk factors for large volume prostate

	*N* (%)	Odds ratio (95%CI)	*P* value	Model ^a^Odds ratio (95%CI)	*P* value	N (%)	Odds ratio (95%CI)	*P* value	Model ^b^ Odds ratio (95%CI)	*P* value	N (%)	Odds ratio (95%CI)	*P* value	Model ^b^ Odds ratio (95%CI)	*P* value
		Total				Age < *70*					Age* ≥ 70*				
TPV > 60 mL															
Normal	126 (49.03%)	Ref		Ref		45 (36.00%)	Ref		Ref		81 (61.36%)	Ref		Ref	
Pre-diabetic	148 (60.41%)	1.59 (1.11, 2.26)	0.011	1.39 (0.96, 2.02)	0.083	53 (55.21%)	2.19 (1.27, 3.77)	0.005	2.27 (1.29, 4.00)	0.004	95 (63.76%)	1.11 (0.68, 1.80)	0.679	0.99 (0.60, 1.64)	0.966
Diabetic	115 (73.25%)	2.85 (1.85, 4.38)	0.000	2.50 (1.57, 3.99)	0.000	35 (70.00%)	4.15 (2.05, 8.41)	0.000	4.74 (2.18, 10.30)	0.000	80 (74.77%)	1.87 (1.07, 3.26)	0.029	1.66 (0.91, 3.02)	0.100
TZV > 30 mL															
Normal	142 (55.25%)	Ref		Ref		48 (38.40%)	Ref		Ref		94 (71.21%)	Ref		Ref	
Pre-diabetic	165 (67.35%)	1.67 (1.16, 2.40)	0.006	1.39 (0.94, 2.05)	0.096	62 (64.58%)	2.93 (1.68, 5.08)	0.000	3.19 (1.78, 5.72)	0.000	103 (69.13%)	0.91 (0.54, 1.51)	0.703	0.71 (0.41, 1.23)	0.219
Diabetic	121 (77.07%)	2.72 (1.74, 4.25)	0.000	2.43 (1.48, 3.99)	0.000	38 (76.00%)	5.08 (2.42, 10.67)	0.000	6.16 (2.70, 14.06)	0.000	83 (77.57%)	1.40 (0.78, 2.52)	0.266	1.20 (0.62, 2.31)	0.588
TZI > 0.5															
Normal	159 (61.87%)	Ref		Ref		61 (48.80%)	Ref		Ref		98 (74.24%)	Ref		Ref	
Pre-diabetic	183 (74.69%)	1.82 (1.24, 2.67)	0.002	1.51 (1.00, 2.27)	0.049	67 (69.79%)	2.42 (1.39, 4.24)	0.002	2.77 (1.53, 5.02)	0.001	116 (77.85%)	1.22 (0.70, 2.11)	0.479	0.95 (0.53, 1.70)	0.856
Diabetic	124 (78.99%)	2.32 (1.46, 3.67)	0.000	2.02 (1.21, 3.37)	0.007	40 (80.00%)	4.20 (1.93, 9.12)	0.000	5.59 (2.39, 13.29)	0.000	84 (78.50%)	1.27 (0.69, 2.32)	0.442	1.01 (0.52, 2.00)	0.967

^a^The model was adjusted by age, body mass index, total testosterone, total cholesterol, triglyceride, high-density lipoprotein cholesterol and low-density lipoprotein cholesterol

^b^The model was adjusted by body mass index, total testosterone, total cholesterol, triglyceride, high-density lipoprotein cholesterol and low-density lipoprotein cholesterol

## Discussion

Age is considered to be a risk factor associated with prostate volume. Evidence of prostate development with increasing age was established in a community-based cohort [11, 12, 14, 15] and in urological outpatients [13]. It is interesting that some studies have shown that the prostate growth rate gradually decreases after the age of 70. Williams and his colleagues described that the prostate growth rate peaked at 4.15 ± 4.98 mL/year between 56 and 65 years old, and then declined rapidly [14]. In two other studies from China, Shi-Jun Zhang et al. [15] found that the prostate had a relatively stable growth rate in men aged 40–70, while Nailong Cao’s study [13] revealed that the fastest period of prostate growth was between 50 and 69 years old. Our results are in line with these studies. Previous studies suggested that age-related damage to the prostate blood supply and changes in sex steroid hormones may be the main causes of prostate growth with age [19, 20]. However, the mechanism by which the prostate growth rate declines during at advanced ages is still unclear.

Diabetes is also a common disease in middle-aged and elderly patients. Years ago, Bourke and Griffin had already mentioned the possible relationship between diabetes and BPH. They found that the prevalence of diabetes was high among those who needed a prostate surgery intervention. They proposed a hypothesis that there was a link between diabetes and the development of BPH [21]. Some similar observations were made in recent years. A study of men over 60 years old from China showed an increase in prostate size in diabetic patients [5]. Another Japanese study demonstrated that high prostate volume in patients with BPH is positively associated with a diabetes diagnosis, and the presence of diabetes was associated with static and dynamic components of BPH [22]. These findings suggested that abnormal glucose homeostasis potentially influences prostate growth, which was similar to the associations observed in Hammarsten’s study [6]. However, few studies have focused on the effect of abnormal glucose homeostasis on prostate growth at different ages, especially the effect of prediabetes on BPH.

It is worth discussing an interesting finding revealed by our results: the patients with abnormal glucose homeostasis had a faster rate of prostate growth before the age of 70 and quickly reached a larger volume capacity, while among men 70 years of age or older, the prostate size no longer increases significantly. Additionally, considering the correlation between obesity and diabetes mellitus, and the fact that a single unit change in the continuous measurement of BMI is unlikely significant clinical impact, we further categorized BMI for a subgroup analysis. However, the results show that only age modified the effect of different blood sugar status on prostate growth. We propose a hypothesis that each male's prostate size may have a threshold. When prostate cells gradually begin to proliferate, the volume rapidly reaches a higher level under the promotion of certain factors such as age, testosterone, or glycemic status. The closer men are to their individual thresholds, the slower the growth rate. This is consistent with what has been found in a previous longitudinal community-based study [23], which showed a negative correlation between TPV growth rate and baseline TPV.

Insulin resistance and secondary hyperinsulinemia [24] may play an important etiological role. Nandeesha et al. [25] found that fasting plasma insulin was an independent risk factor for prostate volume increase in 50 patients with symptomatic BPH and 38 control patients. Other researchers also observed a significant correlation between serum insulin levels and annual prostate or transition zone growth rates [17, 26]. Due to its structural similarity to IGF, insulin binds to the IGF receptor and enters prostate cells, causing the receptor to activate, thereby inducing cell growth and proliferation [27].

Another substantial strength of the present study is that we observed the effect of prediabetes on prostate development. In recent years, prediabetes has been considered a high risk factor for diabetes transformation and has received increasing attention. It is estimated that there were approximately 388 million individuals with prediabetes in China, and the International Diabetes Association predicts that more than 470 million people will have prediabetes by 2030 [9]. Large prospective studies have demonstrated that prediabetes is associated with an increased risk of multiple cardiovascular disease, and the results strongly support the important public health implications of the lower critical point of prediabetes proposed in the 2003 ADA guidelines [28]. Previous studies have focused on the diabetes phase, and there have been few studies on prediabetes. Our results suggested that prediabetes also affects prostate volume and growth rate.

There are certain limitations to the present study. First, this was a cross-sectional study, and determining causal relationships was not possible. The prostate parameters and blood glucose levels were taken only at a single time point. The best way to monitor changes in prostate growth and blood glucose levels is to conduct a longitudinal study in which all participants can be measured and tracked for years. Second, this was a single institution study and the populations selected for these analyses were those individuals who needed surgical intervention. Moreover, 5ARI treatment for more than 2 years can significantly reduce TPV and TZV. Considering that the main research direction of our study was the correlation between the status of glucose homeostasis and prostate size and that treatment with ARI has a certain influence on prostate morphology, we finally excluded patients on 5-a reductase. This likely means that the men selected for this study had a more severe BPH and larger prostate size than the general population of men with BPH. This could have impacted the results, causing a selection bias. Meanwhile, the patient's complete history of hypoglycemic drug uses was lacking in the medical history we obtained, which could be an important confounder, as some medications, such as insulin, are associated with prostate cell growth [29], while other medications, such as metformin, are hypothesized to inhibit prostate cell growth [30]. Further multicenter studies examining the relationship between blood glucose control and prostate growth in patients who are conservatively treated with hypoglycemic drugs are warranted.

## Conclusion

In conclusion, among patients undergoing TURP, prostate volume increases with age. In patients with a normoglycemic state, the prostate growth rate is relatively stable, and the prostate grows to a larger volume after the age of 70. The prostate growth rate was faster in the men with prediabetes and diabetes, and the prostate reached a larger size in the men younger than 70 years old; among the men ≥ 70 years, prostate was significantly slower. Further prospective longitudinal cohort studies in the general population, which include repeated measures of prostate size and ongoing monitoring blood glucose control or medication, are needed to confirm our findings.

## Electronic supplementary material

Below is the link to the electronic supplementary material.

Supplementary file1 (DOCX 16 kb)

Supplementary file2 (DOCX 15 kb)
