# Supplementary material for: The relationship between glucose homeostasis status and prostate size in aging Chinese males with benign prostatic hyperplasia
Source: World J Urol. 2020 Jan 21;38(11):2923–31. doi: 10.1007/s00345-020-03084-4 (PMC7644519; doi:10.1007/s00345-020-03084-4)
Supplement: Supplementary file 1 — Supplementary file1 (DOCX 16 kb) [file 345_2020_3084_MOESM1_ESM.docx]

**Supplementary table 1. The logistic regression analysis of different glucose status and large volume prostate in BMI subgroup**

|  | N(%) | Odds ratio  (95%CI) | *P* value | Model ^†^  Odds ratio  (95%CI) | *P* value | N(%) | Odds ratio  (95%CI) | *P* value | Model ^†^  Odds ratio  (95%CI) | *P* value |
| --- | --- | --- | --- | --- | --- | --- | --- | --- | --- | --- |
|  |  | BMI < 24 | | | |  | BMI *≥* 24 | | | |
| **TPV>60ml** |  |  |  |  |  |  |  |  |  |  |
| Normal | 66 (47.48%) | Ref |  | Ref |  | 60 (50.85%) | Ref |  | Ref |  |
| Pre-diabetic | 78 (62.90%) | 1.88 (1.15, 3.07) | 0.013 | 1.61 (0.96, 2.70) | 0.074 | 70 (57.85%) | 1.33 (0.80, 2.21) | 0.278 | 1.18 (0.69, 2.03) | 0.549 |
| Diabetic | 44 (74.58%) | 3.24 (1.65, 6.37) | 0.001 | 2.65 (1.31, 5.36) | 0.007 | 71 (72.45%) | 2.54 (1.44, 4.50) | 0.001 | 2.25 (1.20, 4.25) | 0.012 |
| **TZV>30ml** |  |  |  |  |  |  |  |  |  |  |
| Normal | 81 (58.27%) | Ref |  | Ref |  | 61 (51.69%) | Ref |  | Ref |  |
| Pre-diabetic | 85 (68.55%) | 1.56 (0.94, 2.59) | 0.086 | 1.29 (0.75, 2.22) | 0.364 | 80 (66.12%) | 1.82 (1.08, 3.07) | 0.024 | 1.49 (0.85, 2.59) | 0.164 |
| Diabetic | 47 (79.66%) | 2.81 (1.37, 5.75) | 0.005 | 2.22 (1.02, 4.81) | 0.044 | 74 (75.51%) | 2.88 (1.61, 5.17) | 0.000 | 2.34 (1.22, 4.49) | 0.011 |
| **TZI>0.5** |  |  |  |  |  |  |  |  |  |  |
| Normal | 91 (65.47%) | Ref |  | Ref |  | 68 (57.63%) | Ref |  | Ref |  |
| Pre-diabetic | 93 (75.00%) | 1.58 (0.93, 2.71) | 0.093 | 1.29 (0.73, 2.30) | 0.378 | 90 (74.38%) | 2.14 (1.24, 3.69) | 0.007 | 1.70 (0.94, 3.05) | 0.078 |
| Diabetic | 50 (84.75%) | 2.93 (1.33, 6.46) | 0.008 | 2.43 (1.03, 5.73) | 0.042 | 74 (75.51%) | 2.27 (1.26, 4.08) | 0.006 | 1.66 (0.86, 3.23) | 0.133 |

^†^The model was adjusted by age, total testosterone, total cholesterol, triglyceride, high-density lipoprotein cholesterol and low-density lipoprotein cholesterol.
