# Supplementary material for: The relationship between glucose homeostasis status and prostate size in aging Chinese males with benign prostatic hyperplasia
Source: World J Urol. 2020 Jan 21;38(11):2923–31. doi: 10.1007/s00345-020-03084-4 (PMC7644519; doi:10.1007/s00345-020-03084-4)
Supplement: Supplementary file 2 — Supplementary file2 (DOCX 15 kb) [file 345_2020_3084_MOESM2_ESM.docx]

**Supplement Table 2**

| **Variables** | **Total**  **(n=659)** | **Normal**  **(n=257, 39.0% )** | **Pre-diabetic (n=245, 37.2%)** | **Diabetic**  **(n=157, 23.8%)** | **P value** |
| --- | --- | --- | --- | --- | --- |
| PSA (ng/ml) | 4.92 (2.50-9.93) | 3.96 (2.16-8.54) | 5.26 (2.93-10.74) | 5.39 (2.70-10.81) | 0.003 |
| IPSS ( score) | 23 (20-26) | 23 (20-26) | 23 (20-26) | 23 (20-26) | 0.806 |
| QOL ( score) | 5 (5-6) | 5 (5-6) | 5 (5-6) | 5 (5-6) | 0.731 |
| AUR ( frequency) | 0(0-1) | 0 (0-1) | 1 (0-1) | 0 (0-1) | 0.237 |
| Qmax ( ml/s) | 5.75 (3.60-8.90) | 6.10 (3.70-8.95) | 5.80 (3.93-9.00) | 4.90 (2.80-8.40) | 0.194 |
| PuraMax ( cmH2O) | 88 (71-107) | 90 (74-110) | 88 (70-107) | 85 (69-103) | 0.167 |
